# Stroke Risks in Primary Aldosteronism with Different Treatments: A Systematic Review and Meta-Analysis

**DOI:** 10.3390/jcdd9090300

**Published:** 2022-09-08

**Authors:** Ningjing Qian, Jingmiao Xu, Yaping Wang

**Affiliations:** 1 Department of Cardiology, The Second Affiliated Hospital, Zhejiang University School of Medicine, Hangzhou 310009, China; 2 Cardiovascular Key Lab of Zhejiang Province, Hangzhou 310009, China

**Keywords:** primary aldosteronism, stroke, adrenalectomy, meta-analysis

## Abstract

Background: Primary aldosteronism (PA) is a common cause of secondary hypertension and confers a higher risk of stroke. The treatment strategies of PA mainly include medical and adrenalectomy treatment, while there is still no solid conclusion on how these two different treatment strategies mitigate the detrimental effect of PA on stroke. Methods: PubMed, Embase, and Cochrane Library were searched for studies comparing stroke events in patients with PA receiving medical treatment versus adrenalectomy treatment published up to 19 March 2022, including patients with essential hypertension as a control group. We used either fixed or random effect models according to the heterogeneities. Sensitivity analysis was conducted by deleting each study one at a time. Results: We reviewed 201 articles, and three studies met the final criteria, including 3244 PA patients with medical treatment, 1611 PA patients with adrenalectomy treatment, and 20,568 EH patients. Patients with PA post adrenalectomy were observed with a significantly decreased risk of stroke compared to patients receiving medical treatment (OR: 0.57, 95% CI: 0.35–0.93, *p* = 0.03), and with no difference when compared to patients with essential hypertension. Patients with PA receiving medical treatment were still observed with higher stroke risks (OR: 1.88, 95% CI: 1.68–2.11, *p* < 0.00001) than patients with essential hypertension. Conclusion: PA is a critical modifiable risk factor for stroke. Adrenalectomy has a superior performance in the mitigation of stroke risks among patients with PA.

## 1. Introduction

Primary aldosteronism (PA) is an autonomous aldosterone secretion syndrome independent of renin, of which the main clinical manifestations are hypertension, electrolyte imbalance, and volume expansion [1,2]. The prevalence of PA is estimated to be 5% to 25% among patients with hypertension and higher among patients with resistant hypertension, as reported [2,3,4,5]. A recent meta-analysis of 31 studies from different regions showed that patients with PA faced more than twice the risk of stroke, compared with those with essential hypertension (EH) [6]. As stroke leads to the growing burden of deaths and disability globally, the prevention of stroke has been an increasingly serious challenge for public health systems worldwide [7]. Consensus is now solid that full awareness and improved management of risk factors could be the most efficient way for the public to tackle the challenge [8]. Therefore, it is urgent to give overdue recognition to the importance of PA in stroke prevention.

The current recommended treatment for PA mainly includes surgical adrenalectomy and medical treatment with mineralocorticoid receptor (MR) antagonists based on PA subtype classifications and patient preference [2]. Given that PA confers a higher risk of stroke, active interventions to prevent PA could be beneficial. However, there is inadequate evidence to indicate whether different treatment strategies could mitigate the detrimental effect of PA on stroke. Therefore, we conducted this meta-analysis to systematically compare the long-term impact of surgical and medical treatment on stroke outcomes among patients with PA.

## 2. Materials and Methods

### 2.1. Search Strategy and Selection Criteria

This meta-analysis was conducted in accordance with the Preferred Reporting Items for Systematic Reviews and Meta-Analyses (PRISMA) statement [9]. To identify all eligible studies, we searched PubMed, Embase, and Cochrane Library using keywords and Medical Subject Headings (MeSH) terms related to PA including ‘primary aldosteronism’, ‘hyperaldosteronism’, ‘aldosteronism’, and ‘stroke’ from the database inception up to 19 March 2022. The studies were assessed independently by two authors according to the following criteria: inclusion of patients with PA; inclusion in two treatment groups, namely patients with PA receiving surgical adrenalectomy and medical treatment, and patients with EH as a control group; inclusion of stroke as an outcome variable; limited to human studies. Only the most recent publication data were selected for duplicate reporting.

Potentially relevant articles were first identified at the title or abstract level of the searched studies against the predefined inclusion criteria by two independent reviewers (N.J.Q. and J.M.X.). The full articles of potentially relevant studies were then further retrieved and evaluated. Any disagreements were solved with discussion or comments from a third reviewer (Y.P.W.). Case reports, conference abstracts, and systematic reviews were excluded.

### 2.2. Data Extraction and Quality Assessment

For the selected studies, two independent reviewers (N.J.Q. and J.M.X.) extracted data using the same template on the data spreadsheets. The following data were extracted: author, journal, year of publication, location of the study group, study design, sample size, population characteristics, treatment strategies, duration of follow-up and data of the desired outcome.

The Risk of Bias in Non-Randomized Studies of Interventions (ROBINS-I) [10] tool was used to evaluate the quality of the selected studies, scoring the risk of bias according to the following domains: ‘Confounding’, ‘Selection’, ‘Classification of intervention’, ‘Deviation from intervention’, ‘Missing data’, ‘Measurement of outcomes’ and ‘Selection of repeated results’. The score of each domain ranges from 0–4, with ‘No information’ (0), ‘Low’ (1—low risk of bias), ‘Moderate’ (2—moderate risk of bias), ‘Serious’ (3—serious risk of bias) and ‘Critical’ (4—critical risk of bias). The study with low risk of bias at most domains would be graded as high quality.

### 2.3. Outcomes of Interest

Efficacy outcome of interest was the incident stroke risk among PA patients with various treatment strategies, namely surgical treatment versus medical treatment. The incident stroke risk was further compared between PA patients with surgical treatment versus matched EH patients, and between PA patients with medical treatment versus matched EH patients.

### 2.4. Statistical Analysis

Extracted data from a standardized data form were pooled in RevMan 5.3 (The Cochran Collaboration, The Nordic Cochrane Centre, Copenhagen, Denmark). The Mantel–Haenszel test was used to calculate pooled odds ratios (OR) with corresponding 95% confidence intervals (CIs). The I-square (I2) test was performed to assess statistical heterogeneity among the included studies. If severe heterogeneity was presented (I2 > 50%), the random effect models were assigned; otherwise, the fixed effect models were used [11]. Moreover, sensitivity analysis was conducted by deleting each study to evaluate the robustness of our results. Funnel plot asymmetry analysis was not performed because the number of studies included in our meta-analysis was <10.

## 3. Results

### 3.1. Included Studies

**Figure 1:**
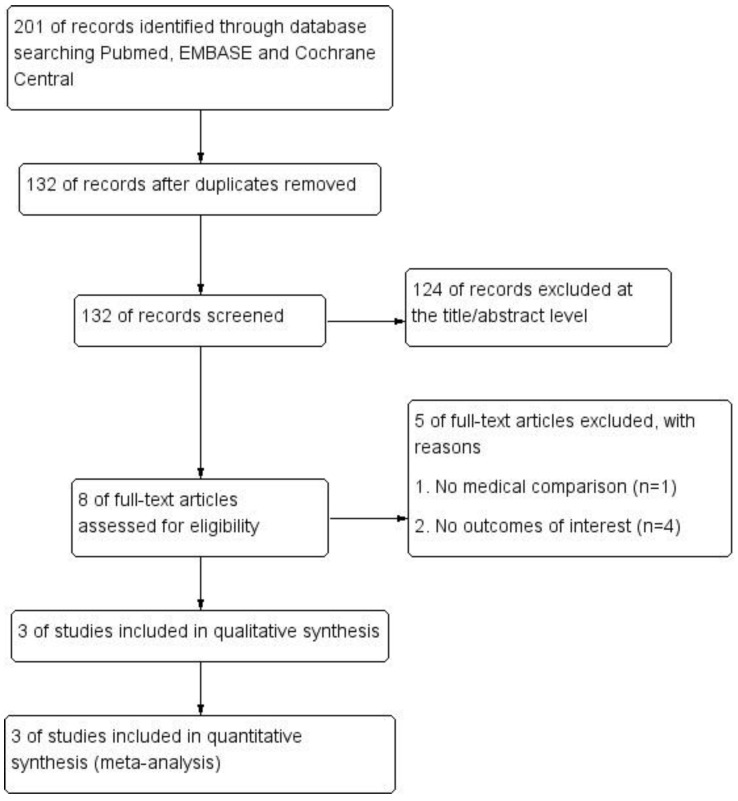
Flow chart of literature search and study selection.

The conducted search yielded a total of 201 articles, of which 69 articles were removed for duplicate reports. After further evaluation for eligibility at the title or abstract level, 124 articles were excluded. We screened the full-text of the remaining eight articles and excluded one due to a lack of a medical treatment comparison group, and four for a lack of an outcomes of interest. Finally, three studies met the inclusion criteria and were included in the meta-analysis [12,13,14], as shown in Figure 1. Table 1 provides the characteristics of the included studies. These studies were conducted in different countries or regions, including Korea, Taiwan and Italy. Moreover, the three studies were all retrospective studies. In brief, a total of 4855 patients with PA, including 3244 PA patients with medical treatment and 1611 PA patients with surgical adrenalectomy treatment, and 20,568 EH patients were incorporated into the final analysis. In the three included studies, the duration of the follow-up varied from one year and up to thirteen years. The mean age was 49.85 ± 13.47 years for PA patients and 49.92 ± 13.46 years for EH patients. Several potential risk factors provided by the original studies, including gender, diabetes, dyslipidemia, and chronic kidney disease, are further summarized in Table 1.

### 3.2. Risk of Bias Assessment

The quality of the included studies was evaluated by two independent reviewers (N.J.Q. and J.M.X.). All three included studies had overall risks of bias at a moderate level as assessed by the ROBINS-I tool. Specific scores of each domain are described in Table 2.

### 3.3. PA Patients Receiving Surgical Adrenalectomy Treatment Compared to the PA Patients Receiving Medical Treatment

**Figure 2:**
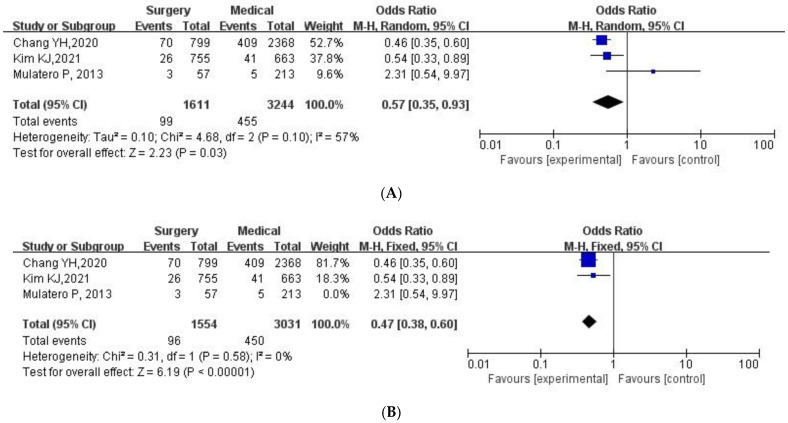
Forest plots of stroke in PA patients receiving surgical adrenalectomy treatment vs. medical treatment. PA, primary aldosteronism. Forest plots for the random effects model (**A**) and fixed effects model (**B**). The blue squares represent the results of individual studies and the black diamonds represent the combined results of total studies in the model.

A total of 3244 PA patients receiving medical treatment and 1611 PA patients receiving surgical adrenalectomy treatment were available for analysis. The risk of stroke was significantly lower in PA patients receiving surgical adrenalectomy treatment compared to PA patients receiving medical treatment (OR: 0.57, 95% CI: 0.35–0.93, *p* = 0.03) in the random effects model, as seen in Figure 2A. Sensitivity analysis was assigned to identify the source of heterogeneity since the heterogeneity of the included studies was moderate (I2 = 57%). When we excluded the data from the study of Mulatero et al. [14] during the sensitivity analysis, I2 decreased to 0% and the difference in stroke risk between the two groups became more significantly apparent (OR: 0.47, 95% CI: 0.38–0.60, *p* < 0.00001) Figure 2B.

### 3.4. PA Patients Receiving Medical Treatment Compared to the EH Patients

**Figure 3:**
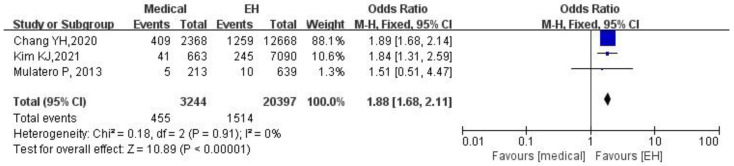
Forest plot of stroke in PA patients receiving medical treatment vs. EH patients. EH, essential hypertension; PA, primary aldosteronism. The blue squares represent the results of individual studies and the black diamonds represent the combined results of total studies in the model.

A total of 3244 PA patients receiving medical treatment and 20,397 EH patients were available for analysis. PA patients receiving medical treatment were significantly associated with an increased risk of stroke compared to EH patients (OR: 1.88, 95% CI: 1.68–2.11, *p* <  0.00001) in the fixed effects model, as shown in Figure 3. Heterogeneity was low (I2 = 0%). Sensitivity analysis by literature analysis and a case-by-case elimination were carried out to check the robustness of our obtained results.

### 3.5. PA Patients Receiving Surgical Adrenalectomy Treatment Compared to the EH Patients

**Figure 4:**
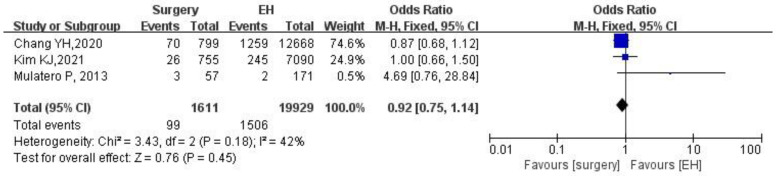
Forest plot of stroke in PA patients receiving surgical adrenalectomy treatment vs. EH patients. EH, essential hypertension; PA, primary aldosteronism. The blue squares represent the results of individual studies and the black diamonds represent the combined results of total studies in the model.

A total of 1611 PA patients receiving surgical adrenalectomy treatment and 19,929 EH patients were available for analysis. In the fixed effect model, patients with PA postsurgical adrenalectomy treatment showed a similar risk of stroke with the EH patients (OR: 0.92, 95% CI: 0.75–1.14, *p* = 0.45), as seen in Figure 4. Heterogeneity was moderate (I2 = 42%) and the robustness of results had been verified by sensitivity analysis.

## 4. Discussion

Our study is the first meta-analysis to investigate the long-term impact of surgical and medical treatment on stroke outcomes among patients with PA and to compare the risks of stroke among patients with PA treated by different therapy strategies and patients with EH. In this meta-analysis including 1611 PA patients receiving surgical adrenalectomy, 3244 PA patients receiving medical treatment, and 20,568 patients with EH, we observed that surgical adrenalectomy treatment was superior in alleviating the increased stroke risk for patients with PA, while the current medical treatment was insufficient to offset the detrimental impact of PA on stroke risk. In addition, the pooled results suggested that for PA patients, postsurgical adrenalectomy treatment had a similar stroke risk with EH patients, and even showed a little bit lower trend (although this was not significant from a statistical standpoint).

The high incidence of stroke has been attributed at least in part to consistent high BP levels caused by PA. In particular, poor BP control is considered a leading risk factor for hemorrhagic stroke [15]. The duration of hypertension is a major contributing factor to stroke events [6,13]. In the previous meta-analysis by Monticone et al., patients with PA (95% lack of treatment information) were observed with a 2.58-fold higher risk of stroke than patients with EH (OR 2.58: 95% CI 1.93–3.45) [6]. In comparison, the risks of stroke for PA patients with either clear medical (OR: 1.88, 95% CI: 1.68–2.11) or surgical adrenalectomy treatment (OR: 0.92, 95% CI: 0.75–1.14) in our study were relatively lower than the result from the study of Monticone et al., which provided evidence that both treatment strategies, at least to some extent, benefit the stroke prevention of PA patients. Studies have shown that 17% to 62% of PA patients receiving adrenalectomy treatment could achieve complete clinical success (normal BP levels without the use of antihypertensive agents) in the long term [16,17,18], and compared to medical treatment, adrenalectomy seems to excel at achieving BP control [19]. This finding would be encouraging for hypertension management in stroke prevention since PA-related hypertension is potentially curable. Moreover, an unsatisfying BP control rate due to poor compliance to antihypertensive agents could be resolved via a single adrenalectomy surgery. Due to better BP control, it is promising to reduce the risk of stroke in PA patients via adrenalectomy.

However, the increased risk of stroke in PA patients can be independent of blood pressure [4,6]. Epidemiological evidence has linked PA with increased prevalence of atrial fibrillation (AF) [20,21]. The results from the meta-analysis of Monticone et al. further suggest that patients with PA are at a 3.52-folder higher risk of AF compared with those with EH [6]. Excessive aldosterone caused AF may further contribute to the incidence of stroke since AF has been identified as a risk factor for stroke [22,23], especially ischemic stroke. Recently, a meta-analysis demonstrated that patients with PA have a lower incidence of new-onset AF post adrenalectomy. In other words, surgical treatment significantly alleviates the detrimental impact of PA on AF events [24], which also supports the results of our study. Consistent with our results of stroke outcomes among patients receiving medical treatment, these patients also remain a high risk of AF. In addition, a study on AF and stroke risks among patients with PA further investigated the time-dependent relationship between these two events and observed that the increased stroke risk was not alleviated with the AF risk reduction over time post medical treatment [12]. This finding reveals that the high stroke risk among patients with PA results from the multiplication of more complicated risk factors. Excessive secretion of aldosterone and continuous stimulation of MR have been shown to have deleterious effects such as inflammation, oxidative stress, fibrosis, and metabolic disorders, on a variety of target organs and systems in the whole body [4,6,25,26,27], which could lead to target organ damages and complications [28] and further collectively account for the increased risk of stroke.

Although surgical adrenalectomy seems to be overwhelmingly promising in the treatment for PA and the prevention of stroke, it is undeniable that there are some shortcomings and deficiencies that surgical adrenalectomy itself is unable to overcome. First of all, surgical adrenalectomy is only recommended for patients with lateralized PA, while for those with bilateral disease or idiopathic hyperaldosteronism, surgical adrenalectomy treatment is inapplicable [29]. In addition, despite the clear benefits of surgical adrenalectomy treatment, there is still a long way to go for the wide application of the therapy [30]. The efficacy of adrenalectomy could highly depend on the preference, experience, and skill of the surgeon. Moreover, the insufficient screening and diagnostic rate of PA should account for this as well. On the one hand, the diagnostic accuracy for PA based on CT scans is limited, while the capability of lateralizing an adrenal venous sampling test operation leads some skill barrier to diagnose PA in real world clinical practice [29,31]. On the other hand, screening for underlying secondary hypertension is often overlooked, especially among patients at a high risk of stroke. Some PA patients might be blocked from accessing the surgical adrenalectomy treatment due to underdiagnosis. The medical treatment, MR antagonists, provides an alternative for PA patients unwilling or unable to receive surgical adrenalectomy [29]. However, there are still a range of issues to be further clarified. Previous studies suggested that MR antagonists work to some extent, though it is not sufficient to completely counteract the deleterious effects of aldosterone. Some had hypothesized that the inadequate blockade of the MR due to inadequate dosage might contribute to unsatisfying treatment outcomes [32]. The dose of MR antagonists needs to be tailored to the individual and titrated to the optimal dose, while underdosage is very common in clinics because of safety concerns. Higher risk of adverse drug effects, for example hyperkalemia, has to be considered if higher dose treatment is performed. There is little agreement on the relationship between dosage and clinical outcomes. Further studies are required for deriving optimal dosage of MR antagonists. Another possible explanation could be that patients receiving medical treatment might have poor medication compliance, which would lead to overestimation of the benefit of adrenalectomy and underestimation of the positive effect of medication on stroke risk reduction. Based on the above, further exploration on therapy strategies, including combined medical and surgical approaches, is necessary.

There are some limitations of this meta-analysis. First of all, the treatment strategy was largely determined based on PA subtype classifications. In this case, the surgical adrenalectomy treatment group patients were almost all those with lateralized PA, while the medical treatment group included those unwilling to receive surgical adrenalectomy and those with bilateral disease or idiopathic hyperaldosteronism. The heterogeneity of the population might misestimate the impact of different treatment strategies on the stroke risk. However, the data of lateralized PA patients with medical treatment were little since adrenalectomy is recommended as a first-line treatment for them [29]. Secondly, it is a pity that the three included studies had not provided adequate information about clinical characteristics such as renin levels, medication compliance, and the dosage of MR antagonists. Monitoring renin levels or other biomarkers would be important to determine whether mineralocorticoid blockade is sufficient. Poor compliance in medication treatment and inadequate dosage might mask the positive effects of drug therapy. Moreover, it would be of interest considering the different dosages when comparing the outcomes among different treatment strategies. Thirdly, the three included studies were all retrospective studies and the follow-up durations varied considerably among the studies. Prospective controlled studies are required to further verify our results.

In conclusion, PA is a critical modifiable though under-recognized risk factor for stroke. Surgical adrenalectomy treatment shows superior positive impacts on the reduction in stroke risks over medical treatment. More treatment strategies should be considered for PA patients for whom adrenalectomy is not feasible, in regard to better risk control.

## Figures and Tables

**Table 1:** Characteristics of the trials included in the meta-analysis.

Reference	Kim KJ, 2021 [12]				Chang YH, 2020 [13]				Mulatero P, 2013 [14]			
	PA		EH	*p* Value	PA		EH	*p* Value	PA		EH	*p* Value
	Medical	Surgery			Medical	Surgery			Medical	Surgery		
Study nature	Retrospective				Retrospective				Retrospective			
Duration of follow-up	Median 5 years				Minimum 1 year, maximum 13 years				Median 12 years			
Number of patients	663	755	7090		2368	799	12,668		213	57	810	
Male, N (%)	657 (46.33)		3285 (46.33)	0.999	1443 (45.6)		5988 (47.3)	0.087	161(59.63)		483(59.63)	1
Age, y	48.83 ± 11.32		48.99 ± 11.34	0.639	50.8 ± 14.5		50.8 ± 14.5	0.992	44 ± 8.5		44 ± 11.4	0.98
Diabetes, N (%)	244 (17.21)		1404 (19.80)	0.005	425(13.4)		1841(14.5)	0.112	11(4.1)		33(4.1)	1
Dyslipidemia, N (%)	579 (40.83)		3506 (49.45)	<0.001	465(14.7)		2007 (15.8)	0.112	74(27,3)		241(29.7)	0.46
CKD, N (%)	137 (9.66)		236 (3.33)	<0.001	70(2.2)		271(2.1)	0.785	N/R		N/R	N/R

CKD, chronic kidney disease; EH, essential hypertension; N/R, not reported; PA, primary aldosteronism.

**Table 2:** Range of overall assessment by study and bias domains.

	Domain 1: Confounding	Domain 2: Selection	Domain 3: Classification of Intervention	Domain 4: Deviation from Interventions	Domain 5: Missing Data	Domain 6: Measurement of Outcomes	Domain 7: Selection of Reported Result	ROBINS-I Overall
Kim KJ, 2021 [12]	2–3	2–3	1	1–2	1	1–2	1	2 Moderate
Chang YH, 2020 [13]	3	1–2	1	1–2	1	2–3	1	2 Moderate
Mulatero P, 2013 [14]	2	2	1	1–2	1–2	2	2	2 Moderate

NOTE. Risk of bias assessment: 0 = No information; 1 = Low; 2 = Moderate; 3 = Serious; 4 = Critical.

## Data Availability

Some or all datasets generated during and/or analyzed during the current study are not publicly available but are available from the corresponding author on reasonable request.
